# The Effect of Parenting and the Parent-Child Relationship on a Child's Cognitive Development: A Literature Review

**DOI:** 10.7759/cureus.30574

**Published:** 2022-10-22

**Authors:** Purva D Lanjekar, Shiv H Joshi, Puja D Lanjekar, Vasant Wagh

**Affiliations:** 1 Community Medicine, Jawaharlal Nehru Medical College, Datta Meghe Institute of Medical Sciences, Wardha, IND

**Keywords:** child/ developmental psychology/ student mental health & parenting, parenting style., child cognition, parent cognitions, cognitive development, parenting

## Abstract

Various studies have been done on subjects such as parenting, parent-child relationships, parenting style, effortful parenting, the concept of parenting, the cognitive development of children, and the cognition of parents. This research is mainly based on parenting practice, child development, and maturation. Children's cognitive development starts in the first year of life and then progresses gradually. Children require positive parenting in painful and different situations. Parenting gives a child the confidence to face crucial, challenging problems. Sensitive parenting and caregiving are required for the child's maturity and cognitive development. Media has been observed to be essential in improving parenting practices. Children exhibit internalizing and externalizing symptoms as a result of harsh, aggressive, and intrusive parenting. According to the data, it is seen that the risk of depression increases in adolescence. The main reason for the rise in depression in children is the non-cooperation and support of mothers and fathers. The risk of depression decreases in children whose mothers and fathers are cooperative and supportive. While family and social stress increase the chances of depression in children, a negative parenting style means children face family and social anxiety. Due to the high level of hostile parenting and low level of positive parenting, they experience stress, peer pressure, and social and family relationship problems. Another excellent term for effortful control parenting is required for children's cognitive development. Parenting is necessary for the regulation of emotions and behavior. There are many problems seen in infancy, early childhood, and late childhood. There are four types of parenting styles seen: authoritative parenting, authoritarian parenting/controlling parenting, permissive parenting/indulgent parenting, and uninvolved parenting/neglecting to parent. Good parenting requires one to know the concept of good parenting, the idea of parenting, the importance of parenting and children's needs, the components of parenting, and the consequences of parenting.

## Introduction and background

Good parenting is a process whereby a parent meets a child's needs according to the cultural standard that changes from generation to generation [1]. Research on parent-child relationships and childhood development increased rapidly during this era; the research is mainly based on parenting practices and child development and maturation. Many studies show that mental and physical stimuli like cognition development, language, social emotion, and children's motor skills in infants and children are very hard to mature or grow. Higher academic performance, income, and socioeconomic development decide childhood growth [2-5]. Parenting is the process of supporting and promoting a child's physical, emotional, mental, and social development. Quality of instructions, animation, cognitive stimulation, physical care, parent-child synchrony, sensitivity, and positive responsiveness are dimensions of parenting and are interrelated with the child's cognitive development. Mainly, research focuses on increasing parental support and responsibility to develop children's cognitive abilities, thus providing sensitive caregiving effects on children's cognitive development. Parenting offers support and care to the child in painful or stressful situations and gives confidence when the child is in non-distressing or non-stressful conditions. Sensitive parenting with young children provides an emotional climate for them. Supporting and sensitive parenting offers children security and confidence; parents promote reasonable regulations and self-initiation in social and non-social experiments. Self-sufficient support and sensitivity, caregiving nature, such as best emotions, lead to early brain maturation and cognitive development in children. Sensitive parenting shows affective and behavioral development in children; it is characterized by responsiveness, positive encouragement for something, giving approval or thanking, stimulation, and lingering [6-8]. Children's health, behavior, development, and style of parenting are always a cause of worry for every parent. In such cases, professional help is required to solve this problem. It is related to family care, youth and parenting support, and children's mental health. The media is exclusively used to increase parenting information and awareness. It enhances parenting skills and decreases depression, anxiety, and stress; it also helps the parents improve the skills of the parent-child relationship and supports them for the benefit of the child's health and well-being [3-5,9]. This research is primarily focused on parenting styles, child cognition, and the concept of parenting.

## Review

Methodology

We searched the PubMed and Google Scholar databases for articles. We identified articles by using words like "parenting style," "child cognition," "parent cognition," "cognitive development," and "parenting." We got 265 results after our search. Out of 265 results, the articles lacking parenting and cognition were excluded. Additional relevant references from these articles were also reviewed.

Parenting and cognition

Zvara BJ et al. [10] observed that for a child to mature, a facilitative environment is required. Children's emotional regulation starts developing in the first year of life and rapidly develops in the coming years. The current and later ways of acting at school age are predicted by the emotional regulations in the preschool period. Children may not develop strong relationships with friends if their mother or father has difficulties regulating their own emotions in front of the children. It is seen that fearful, inhibited boys are affected by sensitive parenting. In contrast, girls with less self-control and fear are not affected by sensitive parenting [11]. Children develop and regulate their emotions when their parents are sensitive and supportive of them. At the same time, it is seen that a greater level of internalizing symptoms like sadness, anxiety, and loneliness, and externalizing symptoms like overreacting, poor impulse control, non-compliance, aggression, and poor relationships with friends are seen in children who do not get proper parenting. It was also noticed that internalizing and externalizing symptoms and problematic peer relationships are associated with harsh, aggressive, or intrusive parenting [10]. Parents play an important role in depression. The risk of depression increases when children's relationships with their mother and father are non-cooperative and lacking support. At the same time, the risk of depression decreases when children's relationships with parents are supportive and collaborative, which is the hallmark of positive parenting. Many studies show that parenting behavior can cause depression. Studies show that youngsters with less warmth, compliance, acceptance or a high level of rejection and criticism from parents develop a poor self-image and negative cognitive style [12,13]. A self-report questionnaire to examine whether a low level of positive parenting contributes to depression via a cognitive vulnerability, including a negative cognitive style, is used. The self-report provides useful, complementary information about parenting behavior. It is seen that positive parenting protects against emotional reactivity and peer pressure but not from non-peer pressure like health, finance, academic, and family problems. At the same time, the adverse events among anxious youth across the transition are limited due to the low positive relationships with parents [13,14]. Recent studies show that it is unclear if family and social stress also cause depression in youngsters. It is also found that negative cognitive style among children, peer stress, and family stress among youngsters are due to the high level of hostile/negative parenting. According to this, it is seen that the maturation of cognitive interpretation in response to social, family, and peer stress during adolescence is resolved by interactive relationships between parents and children. Younger individuals experience stress and peer pressure as well as family stress as a result of low levels of positive parenting and a high level of hostile parenting; this child presents with a negative cognitive style, cognitive vulnerability, depressive symptoms, and depressive episodes. Another type of stress is social stress; it is also due to low levels of positive parenting and a high level of hostile parenting [13,15].

Effortful parenting and control

Positive parenting and effortful control of children are necessary for child cognitive development. Effortful control means suppressing the dominant response to perform the subdominant response. It includes inhibitory control, attention, focusing, and attention shifting. At the same time, components are the ability to initiate or suppress behavior. In children, the self-regulating ability is seen between six and 12 months of age and rises in preschool. Effortful control is also required for children's emotions and behavior regulations [16,17]. This study observed that children who are less able to focus in academic years or socially due to harsh and non-supportive parenting. Due to warm and supportive parenting, a child can regulate their emotions, and they become less frustrated and angry. The ability to initiate or suppress behavior is the central part of effortful control and is related to externalizing problems mainly after 4.5 years of age. Children's external problems are directly proportional to low levels of effortful control. Supportive parenting is linked with early adolescence for self-control and difficult adjustment, while school achievement in children is linked with external behavior and effortful control [16-18]. Much of the research focuses only on the mother-child relationship and not on the role of fathers in parenting. Studies show that fathers also spend time with children, and that time increases gradually. The interaction of the child with the father and mother has differed; the father is always like a friend to the child, while the mother is a caregiver to the child. Fathers encourage the child for emotional stimulation, to solve problems, and to go to the outside world for development and exposure [19]. Hence, the father also plays an essential role in the maturation of exclusive function or condition. While the mother is more related to emotion, she is the source of comfort in stressful stimulation. Maternal parenting is more important; it is associated with the child's effortful control of both current and future stimulation and emotions because the mother uses a positive emotional tone for parenting; therefore, for effortful control of children, positive parenting is necessary. Low levels of effortful control in the child are associated with parental rejection and inconsistency. If parents make positive changes in parenting, then children will demonstrate early learning skills and less problematic behavior. Effortful control affects positive parenting at a younger age for children. At the same time, parenting affects the effortful control of an older generation's life. Hence, it is observed that both paternal and maternal parenting are essential for effortful control and child development [16,20].

Children's cognitive development

Table 1 shows the stages of cognitive development.

**Table 1:** Stages of cognitive development [21, 22]

Stages	Age	Characteristics
Sensorimotor stage (cognitive development during Infancy)	Two years	Incorporation between reflex pattern and child design first (thought), then maintain it, and lastly engulf new changes. Increased understanding of children. The thought process begins, and the child tries to solve the internalized problem.
Preoperative stages (cognitive development during play; preschool age; early childhood age).	Two to seven years	Unsystematic reasoning, development of character representation and language, thought development, mainly egocentrism, animism, etc. Language development is rapid in the pre-conceptual substage (two to four years). The reasoning ability fails to retain a decision or identity related to volume, number, or mass in the perceptual or intuitive substage (four to seven years). There is no flexible thinking, perplexity, or fantasy involved.
Concrete operation stage. (Cognitive development in late childhood stage)	Seven to 12 years	Systemic reasoning develops. Retains the decision and identity of the number, mass, and other properties.
Stage of formal operation	12 years onward	Logic is the reasoning of a hypothetical thought, the evaluation of knowledge, and finding a conclusion to the problem. Think about theories and books and guide them.

According to Piaget's classification, the pre-operational stage is the stage between the ages of two and seven. At this age, children's maturation is a genuinely logical operation. Operation means pliable actions that can interact with each other to solve problems. According to the Piaget theory of cognitive development, older children have more flexible logical operations, while in young children, judgment is dominated by perceptual appearance. According to Muller, children between five and six years of age are surprised to see any weird situation; this is called the primitive identity concept. The primitive idea is the primary milestone. It gives the child reason to think about their gender identity [22,23]. Representational thought is the ability of a child to form mental symbols to represent an object or event that is not present. At the age of two, a child is in the transductive reasoning stage, which means children are influenced by their desires. Children are befuddled by experience and it is difficult for them to remain satisfied with their current way of thinking. Egocentrism in children is the inability to take other points of view. It is not selfless. Instead, it is referred to as "intellectual limitations" [22,23].

Problem and accommodation difficulty during infancy, early childhood, and late childhood

Infancy

Infancy is a time when parents and children adjust quickly to each other for effortful development. In the first year of life, they face feeding problems, digestive discomfort from colic, vomiting, constipation, diarrhea, irregular sleep patterns, and crying. Witnessing the quarrels between mother and father during the baby's physical and mental growth period makes them hostile and unrestrained in the second year of life. Parents report that their child's behavior at this age is stubborn and they have a temper. An infant with autism fails to focus on people's eyes, doesn't show affection when parents leave them, and shows less social interaction. In such cases, children become ignorant, misused, and poorly stimulated [22,24].

Early Childhood

Children in preschool have mobility problems, language-related problems, and immature judgment. Studies and surveys show that a common problem in preschool children is due to representational thinking and fear involving thought and imagination. Other issues are being asocial; using physical and verbal power; being overactive, talkative, angry, whining, showing off, arguing with others, demanding attention, disobedience, and resisting bedtime [22,24].

Late Childhood 

**Figure 1:**
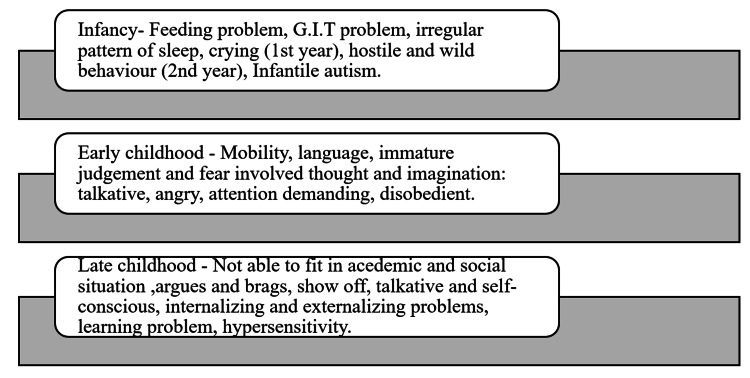
Problems and adjustment issues faced by a child in various phases of childhood. Figure Credits: Purva Lanjekar GIT- Gastrointestinal tract.

A child's experiences in school can determine if he/she "fits in" academically and socially. Some children feel uncomfortable due to low socioeconomic status, casteism, and comparison with friends regarding academic and social matters, leading to mental retardation, physiological disorder, and intellectual problems. The most common problems in this age group are arguing with others, showing off, being talkative, and being self-conscious (most common). School phobia is normal anxiety about school, and it is due to a fear of separation from parents. An internalizing problem pattern is defined as a school phobia with concern, worry, sadness, and diligence. Whereas if hostile behavior is combined with antisocial activity and attitude, this pattern is called an externalizing problem. Dyslexia, reading difficulties, and learning disabilities are examples of learning issues. Children with hyperactivity problems are impulsive and overreact [22,24]. Figure 1 depicts a brief description of the problem and the child's accommodation difficulty.

Parenting styles

There are four types of parenting styles. They are authoritative parenting (warm and firm personality), authoritarian/controlling parenting (minor warm/highly controlling personality), permissive parenting/indulgent parenting (amiable and undemanding), and uninvolved parenting/neglecting to parent (not warm, does not demand anything) [25-28]. Below, Table 2 shows the various parenting styles.

**Table 2:** Parenting styles Source [25-28].

Parenting style	Parenting characteristics	Parental cognition	Child's cognitive development
Authoritative parenting (warm and firm)	Inspires children to be independent but sets some boundaries and limits to control their actions. Listens to the child's point of view and is involved in discussions and debates with children.	Understands the child's point of view, practices sensitive parenting, and scolds a child when required to ensure proper molding of the child's character. Does not use phrases that invoke them, such as "because I said, you should do this."	Healthy development, the self-reliant child within parental limits, guidelines, regulations, and rules.
Authoritarian parenting/controlling Parenting. (a little warm and highly controlling)	The parent takes strict disciplinary action in a restrictive, punitive style. Does not listen to the child's point of view and does not get involved in the child's discussions and debates.	Children should accept their parents' orders without any questions. Does not understand the child's point of view. They always scold the child. Uses phrases that invoke the child, "You did this because I said don't do this." Dominant attitude toward the child	The child will turn out to be rebellious, dependent, and aggressive.
Permissive parenting/Indulgent parenting. (warm, undemanding Parenting)	Parental involvement is passive. The parents do not say no; few boundaries and regulations are not present. If a child is upset, then parents use things (childlike) to make the child feel better.	The parent doesn't participate in active parenting to shape the children's future. If a child asks for anything or asks to do something, then the child depends on parents' advice or permission. Phrases like "you do what you want, no need to ask" are used.	Take your own decision without parental knowledge. The child does not listen to orders.
Uninvolved parenting/ neglected parenting. (this is not "warm parenting; make no demands.")	There is less interaction between parents and children. The parents are uninvolved in the children's needs and don't participate in the experience of the child in school and with peers.	Parents do not want to bother the children. Parents are overwhelmed and self-centered and are engaged with their work and problems. Do not talk to the child. Uses phrases like "I do not care. Do whatever you want. Why should I care about you?"	Impulsive behavior, self-regulation, do not listen to anyone. Does not care about anything.

If both the mother's and father's parenting styles are different, like one parent is authoritarian and the other parent is permissive, then in this condition, they should sit and discuss with each other the situation faced by the child and the child's needs. Cooperative, motivated, and responsible children are a result of the authoritative parenting style, while the uncooperative, immature, irresponsible child is elicited by the uninvolved parenting style. Environmental and behavioral genetic patterns also play a role in children's cognitive development. Parenting style is determined by the mother's and father's behavior, with internalizing problems such as sleep and mood and externalizing problems such as social stress and job difficulty. Individual adolescent characteristics like temperament and personality also play an essential role in parenting [19,25,29].

The concept of parenting

According to research, a survey shows that the cases of crime and violence increase due to a decrease in disciplinary actions in school, divorce, single parenthood, separation of parents, and flight between parents [30]. The cases of drug addiction and homelessness are all due to poverty [31]. Table 3 displays information about the concept of parenting, according to Smetana JG and Hoghughi M [1,32].

**Table 3:** The concept of parenting [1,32].

Good enough parenting concept.	The term "good enough parenting" was coined by Winnicott. When parents are involved in good practical aspects that meet children's needs, it is called "good parenting" and does not need any perfection or alternative parenting style. If parenting is "not good enough" to recognize a child's problem and it gets neglected, then an alternative parenting style is required.
The concept of parenting	Parenting is a group of activities, processes, and relationships between children and their mothers and fathers. Any person who gives a child care, control, and development in any situation is involved in parenting. In parenting, family members, peers, neighbors, teachers, community workers, doctors, and nurses are involved.
Importance of parenting and children's needs.	In the first five years, parenting is required the most because the child depends on parents for physical and emotional control and protection. Good enough parenting gives security to children in difficult situations.
Components of parenting.	Love, care, and commitment. If the child is emotionally unstable from early childhood, it leads to the risk of the development of " affectional psychopathy," the development of insurance attachment, and social and emotional relationship difficulties. 2. Consistent/controllable limit settings 3. Facilitation of development.
The consequences of "not good parenting"	Parental incompetence: Type A: lack of love and care, resulting in an insecure personality, low self-confidence, problematic relationships with friends, marital problems, and parenting difficulties. Type B: Children who are within consistent limits are at risk of future conduct disorders and criminal behavior. Type C: undeveloped, neglected children are at risk of educational failure and are socially handicapped.

Child cognitive development is estimated by the Bayley scales of infant development, Wechsler preschool, and scale of intelligence. At the same time, the sensitivity of the mother's and father's parenting is measured by the observation of the relationship between parents and children [7,33,34]. The interaction between the parent and child and the child's cognitive development is measured by multiple linear regression analyses, adjusting for paternal age, education, depression, infant age, and maternal sensitivity [35].

## Conclusions

Good parenting is how parenting meets the children's needs according to the cultural standards that change from generation to generation. Cognitive development starts in the first year of life and then progresses gradually. Children require positive parenting in painful and different situations. Parenting gives a child the confidence to face crucial, challenging problems. Sensitive parenting and caregiving are required for the child's maturity and cognitive development. Sensitive parenting is required for the proper cognitive development of a child. Proper emotional management is required for proper emotional regulation. Positive parenting helps the child face non-social or social problems. Positive parenting is required for early cognitive development, emotional balance, and the maturation of thought. While negative, hostile parenting leads to depression and social and cultural problems. Parenting styles (authoritative, authoritarian, permissive, and uninvolved parenting) have a psychological effect on a child's behavior.
